# Cable-Driven Parallel Robot with Reconfigurable End Effector Controlled with a Compliant Actuator

**DOI:** 10.3390/s18092765

**Published:** 2018-08-22

**Authors:** Alejandro Rodriguez-Barroso, Roque Saltaren, Gerardo A. Portilla, Juan S. Cely, Marco Carpio

**Affiliations:** 1 Centro de Automática y Robótica, Universidad Politécnica de Madrid, C/José Gutiérrez Abascal 2, 28006 Madrid, Spain; roquejacinto.saltaren@upm.es (R.S.); gera.portilla@gmail.com (G.A.P.); js.cely@upm.es (J.S.C.); mcarpio@ups.edu.ec (M.C.); 2 Grupo de Investigación en Interacción Robótica y Automática, Universidad Politécnica Salesiana, Calle Turuhuayco 3-69 y Calle Vieja, 10150 Cuenca, Ecuador

**Keywords:** cable robot, reconfigurable platform, kinematic redundancy, parallel manipulator

## Abstract

Redundancy in cable-driven parallel robots provides additional degrees of freedom that can be used to achieve different objectives. In this robot, this degree of freedom is used to act on a reconfigurable end effector with one degree of freedom. A compliant actuator actuated by one motor exerts force on both bodies of the platform. Due to the high tension that appears in this cable in comparison with the rest of the cables, an elastic model was developed for solving the kinestostatic and wrench analysis. A linear sensor was used in one branch of this cable mechanism to provide the needed intermediate values. The position of one link of the platform was fixed in order to focus this analysis on the relationship between the cables and the platform’s internal movement. Position values of the reconfigurable end effector were calculated and measured as well as the tension at different regions of the compliant actuator. The theoretical values were compared with dynamic simulations and real prototype results.

## 1. Introduction

Cable-Driven Parallel Robots (CDPRs) are parallel mechanisms whose end effector (EE) is manipulated by using flexible cables coiled in pulleys and winches attached to the rigid frame. Cables can only exert traction and do not resist compression efforts, so it is necessary to provide an opposite force for each cable to maintain its stiffness.

Using cables instead of rigid links with prismatic actuators, such as usual parallel robots, has several advantages due to their wrench-to-weight ratio and simplicity. For example, the Five-hundred-meter Aperture Spherical Telescope (FAST) [1] and the three-dimension moveable camera, SpiderCam [2], have huge workspaces. Further examples are the Robocrane in its six-meter version [3], the Marionet-Crane with sides of 15 m [4], and the assembler of solar panels with a plant size of 15 × 8 m [5]. High speeds and accelerations have been achieved with the configurations provided by using a rigid spine [6,7,8] or without using it as in [9]. Prototypes such as those in [10,11,12,13] have high dexterity due to their parallel configuration and some of them are used in haptic and tele manipulation. This dexterity is used in rehabilitation [14,15,16] and minimally invasive surgeries [17,18]. Additional advantages of using cables in parallel configuration are robots with lower costs, ease of manufacturing, and fast assembly and deployment [19].

As a cable bends without resistance when it is under compressing forces, to maintain the tensile force in a cable, it is needed, whenever possible, to pull the load with the cables located at the opposite side of the end effector. In the case of suspended cable robots, in general, all their cables pull against the gravity force, which acts as a virtual opposite cable. The required number of cables to fully constrain one of those mechanisms with n degrees of freedom (dof) is *n* + 1 [20]. This is needed as the additional degree of redundancy allows for a tension configuration to be set in the cables where all of them are under traction effort. However, in CDPRs with a suspended configuration, the gravity force acts as a virtual cable so in those cases, only n cables are necessary to fully constrain the robot [21].

In this article, a cable-driven parallel robot with a reconfigurable platform was analyzed. These robots have a parallel structure and their end effector is a passive mechanism instead of a rigid body. In [22], Lambert et al. described the fundamental aspects of parallel robots with a reconfigurable platform. In [23], a generalized analysis of this kind of robot was performed by using screws algebra considering the EE as a closed-loop chain.

Knowing the advantages of cable-driven parallel robots, such as their huge translational workspace and their very low inertia, few examples of CDPRs with a reconfigurable end effector have appeared. In [24], a cable-driven robot with reconfigurable end effector was presented. This was a planar haptic device with four dof that provided planar motion. In that study, a geometric and static analysis was developed. In [25], an optimized shape for the reconfigurable end effector for cable-driven suspended robots was proposed with the objective of avoiding obstacles situated on the ground. Some applications of the reconfigured platforms include grasping objects of irregular shape or large volume, such as the first reconfigurable platform robot presented in [26], micro-positioning and haptic devices [23], or end effectors able to avoid singular postures within their workspace [27].

This article focuses on the behavior of those cables actuated by one single actuator that acts on two different bodies. This type of cable, which is able to exert its tension over two bodies at the same time, can be used in CDPRs with a reconfigurable end effector in order to use one degree of actuation over the one degree of freedom of the reconfigurable end effector. This is useful when there is a desire to use one or several degrees of actuation of a redundant CDPR to impose internal motion in the reconfigurable end effector. It can be used in any CDPR in whose end effectors it is desired to add additional degrees of freedom that want to be controlled by using the current degree of actuation. For instance, one robot with eight degrees of actuation and an end effector with seven degrees of freedom (six dof for the rigid body of one body and one dof for the degree of freedom of the second body) used to be controlled by attaching some cables to one body and the rest of the cables to the second body. This method cannot control the six dof of each body; only the dof of the whole reconfigurable end effector can be controlled. This method, already used in [24,25], is interesting because it exerts an equivalent quantity of effort to both end effectors; however, in some poses and orientations, their internal degree of freedom restricts the desired movement of one of the bodies of the end effector due to the appearance of undesired internal movements in the entire platform.

**Figure 1:**
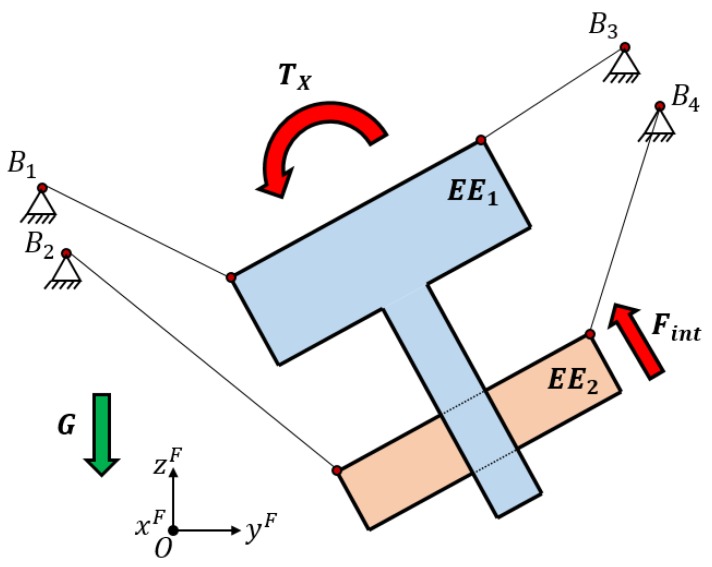
Schematic description of a planar suspended cable-driven parallel robot (CDPR) with reconfigurable end effector (EE) actuated by simple cables. It is seen that when a torque is imposed on the entire end effector, it appears as an internal force in the second body of the end effector, making the control of all degrees of freedom (dof) of one of the bodies difficult.

As seen in the example shown in Figure 1, a planar suspended CDPR with a reconfigurable end effector was actuated by using four independent cables. The second body could only slide along the first body. Two of the cables controlled two dof of the first body while the other two cables controlled the second end effector. As each body had three dof in this planar case, they were under-actuated when they were considered individually. The entire end effector that was composed by the two bodies had four degrees of freedom (three dof of one rigid body and one dof of the available movement of the second body) and four degrees of actuation. However, the movements of the entire end effector were influenced by the internal movement between the two bodies. In the example of Figure 1, there was a desire to obtain a torque in the x-axis of the entire end effector; however, it appeared as an internal force between the two bodies because the cable that is attached to $B_{4}$ has to exert tension to impose the desired torque.

**Figure 2:**
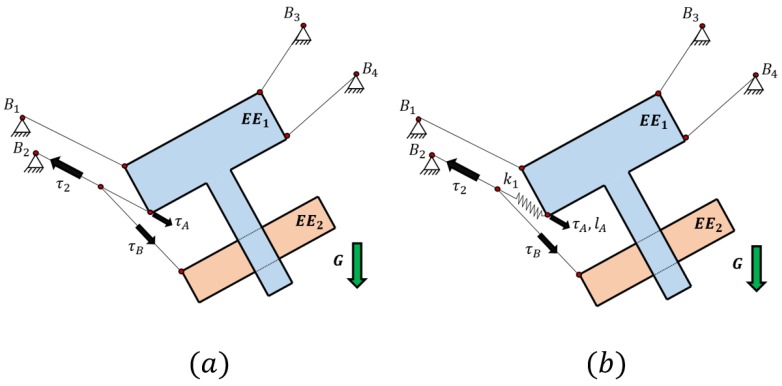
(**a**) Example of a cable acting on the two bodies of the end effector directly. In this case, the first body can be controlled in its three dof by using the four actuated cables. However, if the cable is not elastic, the second body cannot have its movement controlled as it is dependent on the position of the first body with respect to the anchor $B_{4}$; (**b**) Example of a cable acting on the two bodies of the end effector. The elongation of the first branch of the cable can modified by using a spring ($k_{1}$). In this way, the relative position of the second body with respect to the first one can be controlled with the tension $\tau_{2}$. The value of $\tau_{A}$ depends on the position desired for the second body but is compensated for by the three cables that can act on the first end effector in order to have control of the three dof of the first body and the control of the relative position of the second body.

In this article, a mechanical device was proposed together with a method for controlling all the degrees of freedom of one body while the second body is controlled as a secondary task only when the first body is totally controlled. This method was based on the use of a cable that was able to actuate over the two bodies of the end effector at the same time as being actuated by one single actuator. If the elasticity of the cables is neglected by attaching the two branches of the cable directly to both end effectors, it is only possible to set the tension value exerted to one body and the corresponding tension value in the other body. In Figure 2b, the proposed method for controlling all the degrees of freedom of the first body of the end effector without the influence of the internal degrees of freedom of the reconfigurable platform is shown. The second body of the end effector can be controlled with more or less priority with respect to the control of the first body. For instance, if the first body has high priority on its control, the cable that acts on the second body can be tensed with the values allowed by the tension distribution needed by the first body, where the tension boundaries can be set at the value of $\tau_{A}$. However, if the second body needs high priority, it is set at the desired tension value of $\tau_{B}$ without considering whether the cables can provide an appropriate tension distribution in the first body.

By controlling the first body of the end effector, it is possible to apply the well-known control algorithms intended for CDPRs with rigid bodies without the perturbation of the internal movement of the platform as in [28,29,30,31]. However, as seen in Figure 2b, an elastic component needs to be added to the first branch of the cable in order to have an elongation that imposes a motion in the second body. Due to this elongation, an elastic analysis of the cables and springs must be performed.

A usual CDPR with a rigid EE has to determine the tensions applied to the end effector in order to know the wrench applied to it. In the case of this kind of cable, the tension and elongation are considered at the same time, so they need an appropriate sensor system to acquire both signals at the same time. This sensor system is based on a linear position encoder that is able to detect the elongation of the spring of the cable. In this way, by knowing the stiffness of this spring, it is possible to measure the elongation of the first branch of the cable directly and the tension imposed on the first body by the cable in an indirect way.

In addition, the elastic behavior imposed on the robot with the use of springs provides a compliant mechanism in the EE that creates a more secure environment for the interaction between humans and cable-driven robots. As explained in [32], “a compliant actuator will allow deviations from its own equilibrium position depending on the applied external force”. These elastic mechanisms can be upgraded to act as tensegrity manipulators as in [33,34].

### 1.1. Contribution

The contribution of this paper is the analysis of a cable-driven robot with a reconfigurable platform with one of its cables acting in two bodies of the EE at the same time. This is the compliant actuator that acts on two different bodies of the same end effector by considering its position and modifying its tension. Although the first robot with a reconfigurable end effector appeared in 2002 [26], the first robot with this configuration that was actuated by tensed cables appeared in 2017 [25]. Until now, only two references can be found in the literature regarding this last type of cable robot [24,25]. Considering the field of this novel configuration, the main contribution of this article is the consideration of a special cable mechanism that can exert force in two different bodies of the reconfigurable end effector, which is what we call a compliant actuator.

This compliant actuator needs a linear sensor in one of its branches to obtain intermediate information for solving the kinematics and statics of the whole robot. Indeed, the tension of the cable has to be considered to solve the redundancy of the system. For this reason, springs are added in the sensorized branch of the compliant actuator to provide more accurate elongation measures by using a linear sensor. By using the elastic model, it is possible to relate the linear deformation given by the linear sensor with the tension that the sensorized cable exerts to the end effector. Due to the elastic consideration, the compliant actuator can be considered as a tensegrity-based compliant mechanism defined as in [35].

The length value imposed in the actuated part of the compliant actuator is directly used to control the internal dof of the platform by exerting effort in both bodies of the platform. The redundancy of the system is used to compensate for the perturbation added by the sensorized part of the compliant actuator when tension is applied to the compliant actuator by its winch. With this cable mechanism, it is possible to control one degree of freedom while the rest of the cables compensate for the perturbation added by its tension. It is possible to use this mechanism for developing a CDPR with reconfigurable platforms of more than one degree of freedom by using as many compliant actuators as the dof of the end effector.

As can be seen in Figure 2b, by using the sensorized spring it is possible to measure the force applied to the first body by the compliant actuator and also it provides the elongation of this spring with the accuracy of the sensor. However, the position and force in the compliant actuator remain coupled due to the relation between the cable tension and their elongation. This means that, for each geometric configuration of the compliant actuator, there will be a different set of forces that elongate the cables. For this analysis, it is needed to take into account the stiffness of the cables and the forces applied in each of them in order to consider this elongation in the geometric model. Several studies about the stiffness of the mechanism can be found, such as the numerical and experimental estimation of the stiffness model with lumped parameters [36]. When an elasto-geometrical analysis is performed in a cable system, several sources of error appear due to geometric and mechanical uncertainties as demonstrated in [37].

In this article, due to this relation between position and forces, both analyses are made for each sample in the path planning of the movement of the second body of the end effector. The relation between geometry and cable forces is double: on the one hand, it is needed to close the force loop considering the linear elongation and on the other hand it is needed to apply those forces in the line of the cable because their axis lines are the only direction they can exert wrenches. Due to the geometry of this mechanism, an iterative model can be used to achieve the equilibrium of forces in the center of the compliant mechanism considering the information given by the sensorized spring. One iteration calculates the geometry without considering elasticity (geometric resolution), while the wrenches are analyzed in the second part of the iteration considering the stiffness of the cables and the direction of the forces (wrench resolution). The next iteration obtains the geometric position considering the previous equilibrium of forces and elongations. In those experiments, a kinetostatic analysis is performed, so the speed of the movement of the system is slow enough to have time to solve this iteration problem.

### 1.2. Objectives

The objective of this article was to define the kinetostatic analysis of a cable-driven parallel robot with a reconfigurable platform with one degree of freedom considered as the end effector. The analysis was made by fixing one body of this platform to the ground to focus on one compliant actuator. It parts from a winch situated on the upper side of the robot and is split into two different cables. One of these split cables is directly attached to one body of the reconfigurable platform while the other is attached to a spring. This spring connects the terminal of the cable with the other body of the platform. This last side of the compliant actuator with the spring is sensorized by using a linear transducer to measure the length of that spring. This sensor is fixed to the body and its thread goes through the inside of the spring that attaches to the cable as described in Section 3. In this way, the length can be directly measured and the tension of the spring can be obtained through indirect measurements by knowing the stiffness value of the spring and the transducer thread.

A tension study of the cables was also required to have better accuracy in this kinetostatic analysis. This was due to the inner relationship between the cable positions and their forces in mechanisms with elastic cables. A comparison between the theoretical results and simulated and real results are provided in Section 7 and Section 8. We also analyzed the influence of the considered simplifications in the mathematic model in comparison with the simulated and real model to compare the position errors.

As can be seen, to achieve the proposed goals, the use of a linear sensor was considered to obtain intermediate information of the mechanism. Linear sensors have been already used to solve the direct kinetostatics problem in cable-driven parallel robots, such as that in [38], and in rigid links parallel robots, such as that seen in [39]. The use of this linear sensor combined with a spring can provide a measurement of length and tension in one of the branches of the compliant actuators when the spring is modeled with accuracy. This way of measuring provides the necessary information to solve both problems: kinetostatic and tension configuration.

The main advantage of the use of compliant actuators is the possibility of controlling the main end effector with all the actuated cables with all the wrench capability the sole end effector has as a rigid body. The control of the dof of the second body of the reconfigurable end effector can be done by using only one branch of this special cable. When no control is needed in the second body, the first end effector can regain the full control provided by all the actuators. This property is useful if the second body acts, for instance, as a gripper that is only actuated on certain occasions while the main end effector can have all its capabilities the rest of the time.

This cable-driven mechanism with a reconfigurable end effector is being developed on a small scale to analyze the behavior of the compliant actuator controlling the inner dof of the platform. The final objective was to bring those results to a large-scale cable-driven robot that is able to simulate environmental dynamic conditions in humans and humanoid robots. In this way, it is possible to simulate underwater, low-gravity, or other dynamic conditions. The CDPR with a reconfigurable platform is desired for adding one additional degree of freedom in the underwater humanoid that is being tested in the simulator. In this way, the $EE_{1}$ provides six dof in the trunk of the robot while the additional dof can provide dynamics in its hips to simulate underwater torque. Additionally, non-gravity or outer space dynamic simulations can be performed. With the use of the compliant actuator, all of the actuators are situated far from the humanoid to reduce risks.

Other applications that are being developed for this robot include grasping large and irregular objects in very large workspaces by using the advantage of the cables instead of rigid links in the robot structure. Furthermore, the compliant behavior of this compliant actuator allows for this kind of robot to direct human–robot interaction.

## 2. Redundancy Resolution

Cable robots are usually redundantly actuated to increase their workspace to make them robust under faults or for increasing their dexterity [40]. The degree of redundancy of a robot is defined as:

(1) r=m−n

where *n* is the degree of freedom of the mechanism; *m* is the number of actuators; and *r* is the degree of redundancy. When *r* > 0, for a given wrench applied at the *EE*, there exists infinite solutions for the values of tension in the *m* cables. Those solutions are the different tension configurations of the CDPR. This tension distribution can be defined in the following way [41]:

(2) $$\tau =-{\left(A\right)}^{†}W+N\lambda$$

where τ is the m-dimension vector of the tension of the cables; ${\left(A\right)}^{†}$ is the Moore–Penrose pseudoinverse of the non-square wrench matrix of the mechanism; W is the vector of external wrench applied in the *EE*; N is the (*m* × *r*) matrix that defines the null space basis of ***A***; and $\lambda =\left[\lambda_{1},\;\ldots \;\lambda_{r}\right]$ is an arbitrary vector in the null space basis. This vector allows for the selection of any tension distribution in cables (τ) that fulfils the constraints of maximum [42] and minimum [43] tension in each cable.

The left side of Equation (2) is the particular solution of the tension distribution that has a minimum 2-norm. The right side is the homogeneous solution that spans all possible tension configurations. Applying the principle of virtual work and screw theory, a wrench matrix (***A***) is defined as the transpose of the Jacobian matrix of the mechanism [44]:

(3) $$A=\left[\begin{array}{cc} \begin{array}{c} u_{1}\;\\ p_{1}\times u_{1} \end{array} & \begin{array}{cc} \ldots & \begin{array}{c} u_{n}\\ p_{n}\times u_{n} \end{array} \end{array} \end{array}\right]$$

where $u_{i}$ is the unitary vector with the same direction of the force that the *i*-cable exerts to *EE* and $p_{i}$ is the vector of the point of the EE where that force is applied. Using the concept of Screw Theory [45] for simplicity of calculus, the origin of vector $p_{i}$ is considered to be the EE’s center of mass [45].

In [46], a kinestostatic analysis was made of a spatial mechanism of rigid links defined as tensegrity-based with compliant connectors. It had seven actuators and six dof. Moon et al. established the internal energy value for the springs to constrain the *r* = 1 redundant degree of freedom. In this article, redundant dof were not constrained by energy assumptions as in [47], but by the tension of one specific cable related to its linear deformation. This length variation due to cable tension was amplified by using springs with less stiffness than the cable. Those springs were situated in serial between the cables and *EE*. The tension value of one of these springs can be set to a desired value if the system has available degrees of redundancy to do so. One degree of redundancy must be left free to fulfill the restriction of tension boundaries in each cable as stated in [20]. With this assumption, this analysis can be done for any cable-driven, full-constraint robot with *r* > 2 or a suspended kind of *r* > 1 [20,21]. Experiments conducted in this article considered a generic eight cable prototype with an end effector of six degrees of freedom, so *r* = 2 degrees of redundancy were available where one of them was used to ensure that all cables were tensioned, and the other was set to control the cable tension and its related elongation.

This tension value was imposed in the redundancy analysis to obtain a feasible tension configuration. The value of this tension is the force that the compliant actuator exerts on the end effector when the second body of the reconfigurable platform is being controlled.

The elongation of the controlled springs is proportional to the tension force measured in the springs attached to the *EE* ($\tau_{j}^{*})\;\left(j=n+1,\ldots ,\;m-1\right)$:

(4) $$\tau_{j}^{*}=k_{j}\left(l_{j}-l_{0}{\;}_{j}\right)=k_{j}\delta l_{j}$$

where $k_{j}$ is the stiffness of *j* spring and $l_{0}{}_{j}$ is its equilibrium length. The value of $\delta l_{j}\;\left(j=n+1,\;\ldots ,\;m-1\right)$ is set as the necessary condition to obtain the tension configuration of the *m* cables. This tension configuration can be obtained using Equation (2), imposing the values of $\tau_{j}^{*}$ of the *r* − 1 springs whose elongation is set and solving the *m* − 1 equations for the *r* − 1 values of λ and the *n* values of $\tau_{i}\;\left(i=1,\ldots ,\;n\right)$. As mentioned before, we let $\lambda_{r}$ be unconstrained in order to fulfill the restriction of making every cable pull. So, there were *m* equations and *m* + 1 unknown values. This expression, derived from Equation (2), has the form:

(5) $$\left[\begin{array}{c} \tau_{1}\\ \begin{array}{c} \vdots \\ \tau_{n}\\ \begin{array}{c} \begin{array}{c} \tau_{n+1}^{*}\\ \begin{array}{c} \vdots \\ \tau_{m-1}^{*} \end{array} \end{array}\\ \tau_{m} \end{array} \end{array} \end{array}\right]=-{\left(A\right)}^{†}W+N\left[\begin{array}{c} \lambda_{1}\\ \vdots \\ \lambda_{r} \end{array}\right].$$

In Equation (2), there were *m* independent variables from the tension provided by cables plus *r* independent variables from the degrees of redundancy with m equations. In Equation (5), as the *r* − 1 values of $\tau_{j}^{*}$ are set to a desired value, there are only *m* − (*r* − 1) independent variables from the cable tension and *r* for redundancy. Letting there be one degree of redundancy in order to have some freedom of choice in the cable tension configuration avoids configurations where a cable tries to push.

Equation (4) provides $l_{j}$ as the independent variable so that the end effector can be substituted by a mechanism with (*r* − 1) dof. The range of possible values of $l_{j}$ increases with the range of $\tau_{j}^{*}/k_{j}$ as seen in Equation (4).

The *m* actuators of the CDPR, situated in the fixed base, can fully control the (*m* − 1) degrees of freedom of the *EE* considered as a reconfigurable platform. Although some links of the reconfigurable *EE* are under high cable tension for maintaining the imposed equilibrium of Equation (2), there are some links whose tension comes only from the values of the desired $l_{j}$. That tension depends on the stiffness of the closed-loop mechanism of the *EE*.

## 3. Mechanism Description

**Figure 3:**
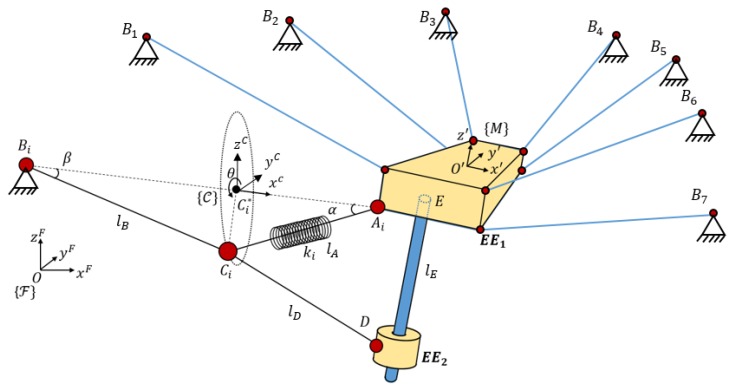
Schematic description of a cable-driven robot with a reconfigurable platform of two bodies ($EE_{1}$ and $EE_{2}$). The compliant actuator that parted from $B_{i}$ to $A_{i}$ and D could control both bodies of the reconfigurable end effector at the same time.

The mechanism is a cable-driven parallel robot with a reconfigurable end effector of one dof. As seen in Figure 3, all cables parted from fixed points where m actuated winches released or collected the cables. Those winches were situated at generic fixed points. In this way, the motors that act on the winches could control the length of all the cables. The analysis of this article focuses on the modeling of a special cable that was able to control both bodies of the end effector, which was the compliant actuator. The compliant actuator parted from $B_{i}$ to $A_{i}$ and D and was controlled by the motorized winch situated in $B_{i}$; however, in $C_{i}$, this cable was divided into two cables that provided mechanical tension to $EE_{1}$ and $EE_{2}$.

A priori, the compliant actuator had one controlled input but two outputs in each body of the platform, which is why a linear sensor was situated between $C_{i}$ and $A_{i}$. This linear sensor provided the elongation of that part of the compliant actuator, which was related to its mechanical tension. In order to amplify the elongation/tension value, a spring was added in series in that cable. The linear sensor passed through the inside of the spring to provide the elongation of the section $\bar{C_{i}A_{i}}$ and so its tension value. This value was obtained by using Equation (4). The length between $B_{i}$ and $C_{i}$ is $l_{B},\;l_{D}$ is the length from $C_{i}$ to D, and $l_{A}$ is the length measured by the linear sensor from $C_{i}$ to $A_{i}$. The value of $l_{B}$ is controlled by a winch, $l_{D}$ is a section of cable with a defined length (when no forces are applied), and $l_{A}$ is the value measured with the linear sensor.

The mechanism designed to be the *EE* of the robot had one dof. The two links of this mechanism were designated as $EE_{1}$ and $EE_{2}.$ The body $EE_{2}$ had a cylindrical articulation (*D*) that could move freely through a linear guide. This linear rod was solidary to $EE_{1}$ with direction z’ and passed through E. $\left\{M\right\}:O^{\prime }-x^{\prime },y^{\prime },z^{\prime }$ is the frame linked in the center of the top face of $EE_{1}$.

The cable $B_{i}C_{i}$ had a spring attached at its end with stiffness $k_{i}$. This spring defined the line $A_{i}C_{i}$, whose length was measured by using the linear position encoder. These types of encoders add a tension in $C_{i}$ with direction to $C_{i}A_{i}$ that was characterized in order to be considered in the model. It also needed to measure the values of the spring stiffness considering them in the function of elongation $C_{i}A_{i}$. This model $k_{i}\left(\|C_{i}A_{i}\|\right)$ can be obtained from a traction test of the spring.

The segment of cables that did not have a spring is modeled as a linear spring in order to consider variations of length due to cable deformation; in addition, the cable was considered without mass.

**Hypothesis** **1.** *Cables have negligible mass. Tension force in cable*$B_{i}C_{i}$*is exerted due to the weight of the body*$EE_{2}$*. To maintain this tension,*$D_{i}$*has to be at a lower height than*$C_{i}$*in the fixed frame*$\left\{F\right\}:O-x^{F},y^{F},z^{F}$:

(6) $${\left(\vec{C_{i}A_{i}}+\vec{A_{i}O^{\prime }}\right)}^{F}\cdot {\hat{k}}^{F}<{\left(\vec{D_{i}E_{i}}+\vec{E_{i}O^{\prime }}\right)}^{F}\cdot {\hat{k}}^{F}.$$

**Figure 4:**
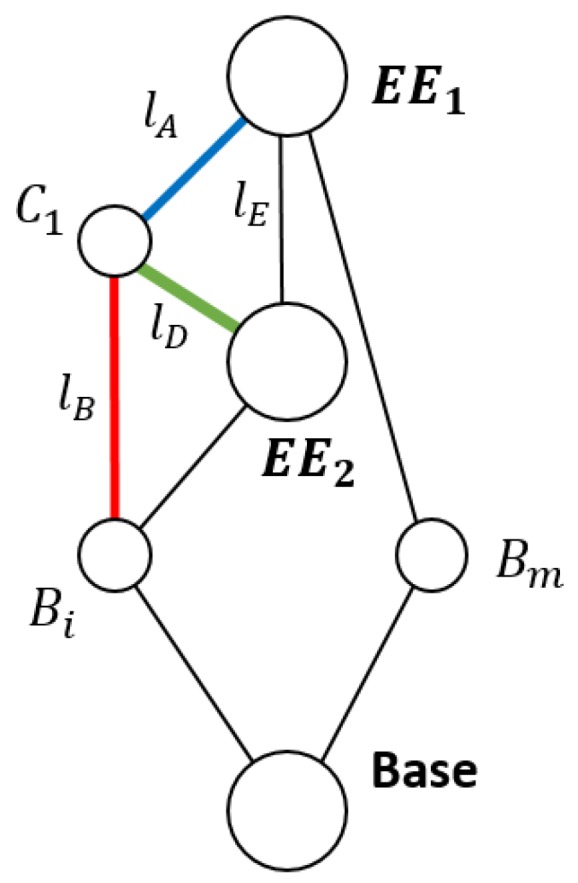
Graphical simplified representation of robot topology. The red line is the $l_{B}$ cable with imposed tension exerted by the actuator; the green line is $l_{D}$ that transmits effort to the second end effector and the blue line $l_{A}$ represents the linear sensor link.

Using Graph Theory and Screw Theory [22], it is possible to represent, after simplification, this mechanism as that seen in Figure 4.

As seen in Figure 4, all parallel legs $Base-B_{i}-EE_{1}$ can be reduced to a unique wrench obtained from the linear combination of all the wrenches of the legs. The (*r* − 1) legs that have an additional end effector are analyzed in the following section.

## 4. Definition of the Problem

This article aimed to find the relation between the length of $l_{B}$ and the distance between the two bodies of the end effector along its degree of freedom restricted by the rod. The compliant actuator length ($l_{B}$) was controlled by a motorized winch fixed in $B_{i}$. Therefore, this problem focuses on the behavior of the compliant actuator. In Section 2, we defined how it is possible to exert tension in the compliant actuator without interference from the resultant wrench applied in $EE_{1}$. Considering that all cables can compensate for the effect of the compliant actuator as seen in Equation (5), it is possible to impose forces and torques values on $EE_{1}$ with independence on the value of tension provided by the compliant actuator. However, in order to set the desired wrench in $EE_{1}$, it is necessary to know the tension value of the compliant actuator. The linear sensor according to Equation (4) provides that measure indirectly. For this analysis, the $EE_{1}$ was considered fixed in space in order to perform a specific analysis of the compliant actuator. This assumption was made by imposing null resultant wrenches in $EE_{1}$.

In order to reach the proposed objectives, a direct kinetostatic problem was formulated in this section. The objective of the analysis was to calculate the length $\left\|\vec{ED}\right\|$, designated with $l_{E},$ that is the distance between the two bodies of the end effector. The known values are the position of the points in the fixed base $B_{i}$ and the position and orientation of $EE_{1}$. That attitude of $EE_{1}$ can be specified with Euler angles or quaternions. The homogeneous transformation matrix from {M} to {F} is:

(7) $$T_{M}^{F}=\;\left[\begin{array}{cc} R_{M}^{F} & \vec{OO^{\prime }}\\ 0 & 1 \end{array}\right]$$

where $R_{M}^{F}$ is the rotation matrix between the two reference frames. The location of the attachment point of the cable to $EE_{1}$: ${\left(\vec{A_{i}O^{\prime }}\right)}^{M}$ is also known, which can be seen from the fixed frame by using

(8) $${\left(\vec{A_{i}O^{\prime }}\right)}^{F}=R_{M}^{F}{\left(\vec{A_{i}O^{\prime }}\right)}^{M}.$$

In the kinetostatic analysis, dynamic efforts are neglected by performing low accelerated movements. Only the weight of *EE* is considered as an external wrench W to be substituted in Equation (2). That wrench is measured from {M}. The value of $l_{E}$ is one of the independent variables and its value will be one of the (*m* − 1) reference values for commanding the robot (*n* values for positioning $EE_{1}$ and one value for setting $l_{E}$). The length $\left\|\vec{A_{i}C_{i}}\right\|$ is known by using a linear encoder so the tension in $A_{i}C_{i}$ can be obtained using Equation (4).

The objective of the process was to obtain the direct kinetostatic resolution. The value of $l_{E}$ was obtained for each value of $l_{B}$, which is the length from B to C. Due to the longitudinal deformation of the cable when tension is exerted and the uncertainty of how the tension of the BC cable is exerted in the AC cable and the CD cable, it is necessary to add a linear sensor to obtain additional information of the length and tension in cable AC.

**Figure 5:**
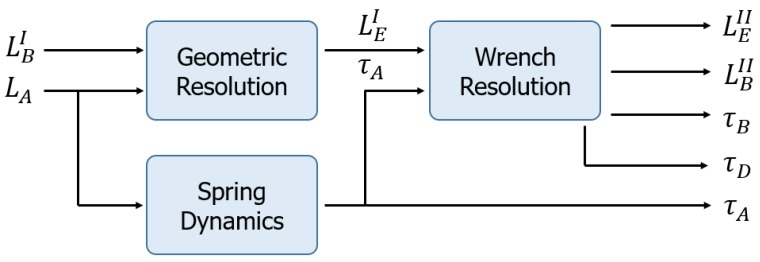
Scheme of the resolution of the kinetostatic of the compliant actuator of the robot.

Cable tension is related to geometric configuration. This is because tension in the cables is exerted only along their axis so they reconfigure themselves to align their geometric axis with the force vectors. This issue demands an iterative resolution that combines the kinetostatic resolution with stability in the tension values in order to converge to the precise solution for each sample of the position of the robot. In Figure 5, the scheme for the resolution of the kinestatic of the compliant actuator of the robot can be seen. A priori, the known values are $l_{B}^{I}$ defined by the actuator and the winch that releases the cable. The value of $l_{A}$ is also known and is directly provided by the linear sensor. Geometric resolution assumes that cables are inextensible and the desired $l_{E}^{I}$ value is obtained as seen in Section 5. However, because we considered deformable cables, we needed to know the tension in each cable to obtain their elongation. This relation is the same as Equation (4) because the cable has been modeled as a linear spring. This resolution is developed in Section 6. Once the updated values of cable length have been obtained, another iteration can be performed to reach more accurate results.

In this analysis, the velocities of the robot were small enough to neglect the dynamic effects and the position and tension changes were very small. Therefore, only one geometric and wrench iteration is solved in each sample, aiming to converge to more precise solutions in the following samples. If the system moves faster or if a more precise solution is needed, more iterations can be done for each value of $l_{B}$.

## 5. Geometric Resolution

The first step of the analysis was to define the geometric place where point $C_{i}$ could be situated. Considering the lengths $l_{B}$ and $\left\|\vec{C_{i}A_{i}}\right\|$, $C_{i}$ was situated at the intersection of the two spheres with those radiuses and centered in $B_{i}$ and $A_{i}$, respectively. This region is a circumference centered in:

(9) $${\left(C_{i}^{*}\right)}^{F}=B_{i}^{F}+\frac{{\left(\vec{B_{i}A_{i}}\right)}^{F}}{\left\|\vec{B_{i}A_{i}}\right\|}\|\vec{B_{i}C_{i}^{*}}\|.$$

The distance between the point where the cable begins ($B_{i}$) and the center of the circumference is:

(10) $$\|\vec{B_{i}C_{i}^{*}}\|=\frac{\left({\left\|\vec{B_{i}A_{i}}\right\|}^{2}-{\left\|\vec{A_{i}C_{i}}\right\|}^{2}+l_{B}^{2}\right)}{2\|\vec{B_{i}A_{i}}\|}.$$

The cable has two deviation angles from the theoretical straight line between $B_{i}$ and $A_{i}$ due to cable tension $\tau_{C}$: angle β in the pulley or winch $B_{i}$ and α in the anchor point of EE. These angles are measured in the plane that contains the triangle $B_{i}C_{i}A_{i}$ and are obtained with:

(11) $$\beta =\cos^{-1}\left(\frac{\left\|\vec{B_{i}C_{i}^{*}}\right\|}{l_{B}}\right)$$

(12) $$\alpha =\cos^{-1}\left(\frac{\left\|\vec{B_{i}A_{i}}\|-\|\vec{B_{i}C_{i}^{*}}\right\|}{\left\|\vec{A_{i}C_{i}^{*}}\right\|}\right).$$

Knowing this, the radius of the circumference is:

(13) $$h=l_{B}\sin\beta =l_{B}\sin\left({cos\;}^{-1}\left(\frac{\left\|\vec{B_{i}C_{i}^{*}}\right\|}{l_{B}}\right)\right)$$

(14) $$h=l_{B}\sqrt{1-{\left(\frac{\left\|\vec{B_{i}C_{i}^{*}\;}\right\|}{l_{B}}\right)}^{2}}.$$

The radius is strictly bigger than zero (h>0). This circumference has high relevance in this analysis so a reference frame $\left\{C\right\}:C^{*}-x^{C},\;y^{C},\;z^{C}$ was defined in order to clarify the analysis. $x^{C}$ has the direction of the line between $B_{i}$ and $A_{i}$; $y^{C}$ is perpendicular to this line and perpendicular to $z^{F}$. The direction and orientation of this frame is defined in Figure 3.

Point ***C*** is situated at a point of that circumference. That position depends on the wrench exerted by $EE_{2}$ in the system and in the length of $\left\|\vec{CD}\right\|$. The position of $C^{C}={\left[C_{X}^{C},\;C_{y}^{C},\;C_{Z}^{C}\right]}^{T}$ with respect to {C} is:

(15) $$C_{x}^{C}=0$$

(16) $$C_{y}^{C}=h\cos\theta$$

(17) $$C_{z}^{C}=h\sin\theta .$$

Point C expressed in {F} is:

(18) $$C_{i}^{F}=T_{C}^{F}C^{C}+C^{*}.^{F}$$

With $T_{C}^{F}$ the transformation matrix from {C} to {F}:

(19) $$T_{C}^{F}=\;\left[\begin{array}{cc} R_{C}^{F}\; & \vec{OC_{i}^{*}}\\ 0 & 1 \end{array}\right].$$

In Equations (16) and (17), the value of θ is not known. Additional information related to $EE_{2}$ is needed to have closed-loop equations. The position of D has to be along the line ED and it is a function of $l_{E}$:

(20) $$D^{F}=D^{F}\left(l_{E}\right)=O^{\prime F}+T_{M}^{F}\left({\left(\vec{O^{\prime }E}\right)}^{M}-l_{E}{\vec{\frac{ED}{\left\|\vec{ED}\right\|}}}^{M}\right).$$

Matrix $T_{M}^{F}$ is defined in Equation (7) and the vertical rod has the direction of ${\vec{z}}^{M}$. The unknown value of θ defines infinite planes Π=Π(θ) defined by the points $B_{i}^{F},\;A_{i}^{F},\;C_{i}^{F}$ and also $C_{i}^{{*}^{F}}$. Formulating it in the Hessian normal form, its normal vector is:

(21) $${\hat{n}}^{C}=-\frac{1}{h}\frac{\partial C^{C}}{\partial \theta }={\left[0,\;\sin\theta ,\;-\cos\theta \right]}^{T}$$

(22) $${\hat{n}}^{F}=T_{C}^{F}n^{c}.$$

**Figure 6:**
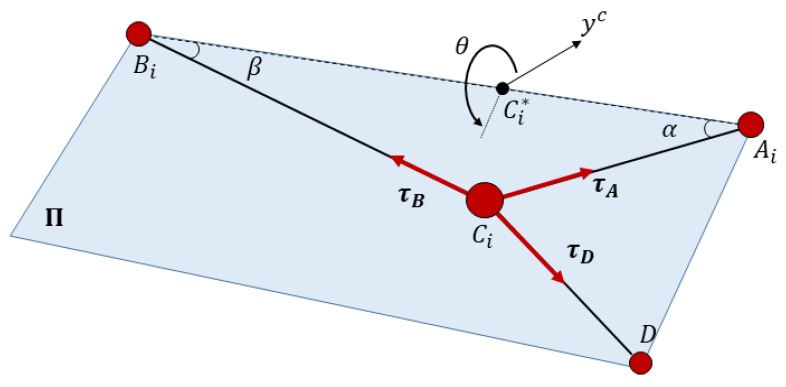
Equilibrium of forces in point $C_{i}$.

**Hypothesis** **2.** *The mass of*$C_{i}$*can be neglected. With this consideration, it is possible to analyze the equilibrium of forces applied in*$C_{i}$*as coplanar vectors as is shown in Figure 6*.

The relation between θ and $l_{E}$ is obtained from the intersection between Π and $l_{E}{\hat{k}}^{M}$:

(23) $$D^{F}\cdot {\hat{n}}^{F}=0.$$

To obtain the isolated value of $l_{E}$, Equation (22) can be rewritten as:

(24) $$D^{F}=E^{F}-l_{E}V^{F}.$$

Combining Equations (23) and (24) and applying a distributive property, we can obtain:

(25) $$E^{F}{\hat{n}}^{F}-l_{E}V^{F}{\hat{n}}^{F}=0$$

(26) $$l_{E}=\frac{E^{F}{\hat{n}}^{F}}{V^{F}{\hat{n}}^{F}}.$$

The operation of Equation (24) needs the vector ${\hat{n}}^{F}$ that defines Π**,** which depends on the unknown angle θ. In order to obtain this, it is necessary to impose the known value of $l_{D}$ on the distance between $C_{i}$ and D.

(27) $$D^{F}-C_{i}^{F}=E^{F}-\frac{E^{F}{\hat{n}}^{F}}{V^{F}{\hat{n}}^{F}}V^{F}-C_{i}^{F}$$

(28) $$\|D^{F}-C_{i}^{F}\|=l_{D}$$

**Figure 7:**
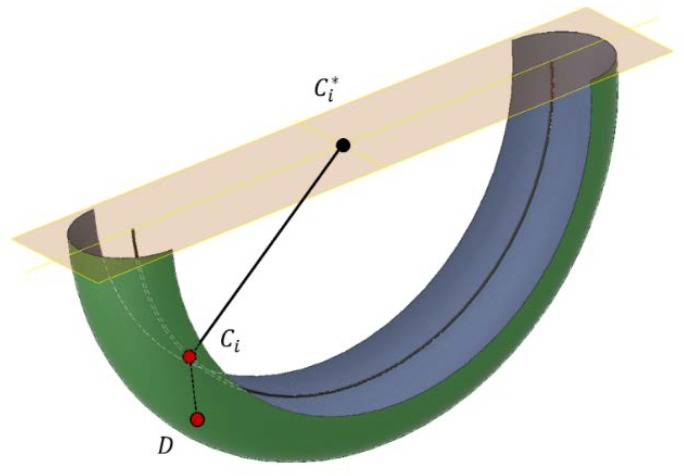
Geometric region of valid existence of point D.

Regarding the position of the linear rod $\vec{E_{i}D_{i}}$, solutions of the direct kinetostatic problem are shown in Figure 7. It is known that $C_{i}$ is situated in a semi-circumference (black line in Figure 7) and point D is in plane Π, which contains points $C_{i}^{F},\;C_{i}^{{*}^{F}},\;D^{F}$, so the geometrical shape of the possible positions of D is a torus of $R=\|\vec{C_{i}C^{*}}\|$ and $r=\|\vec{C_{i}D}\|$. If we impose conditions of positive traction in all of the cables, it can be seen that point $C_{i}$ has to be lower than the horizontal plane that contains $C_{i}^{{*}^{F}}$ to maintain the tensions $\tau_{A}$ and $\tau_{B}$ as was already proposed in Equation (4). In addition, the interior face of the torus corresponded to configurations where $C_{i}$ has to exert effort against the three cables, which is impossible with this mechanical design (unless punctual high dynamics effects are considered).

With the previous boundaries considered as represented in Figure 7, apparently there exists one or two solutions for the direct kinetostatic problem depending on the position and orientation of the vertical rod. However, a third condition is based on the projection of the weight of $E_{2}$, $-m_{2}{\vec{g}}^{F}$ on ${\vec{ED}}^{F}$. This projected tension has to provide a positive value of $\tau_{D}$. A mathematic way to see this condition is:

(29) $${\left(\vec{ED\;}\right)}^{F}\cdot {\left(\vec{C_{i}D}\right)}^{F}>0.$$

$EE_{1}$ is not able to turn more than π/2 rad in $x^{F}$ or in $y^{F}$, so the vector of weight projection on the vertical rod is always from E to D, and Equation (27) is always valid. Considering these three additional restrictions based on the imposition of positive traction, we passed from the consideration of four solutions to have a unique one.

## 6. Wrench Resolution

Once the direct kinetostatic problem is solved by obtaining the value of $l_{D}$ in the function of $l_{B}$, it is possible to obtain the wrenches in the three cables that make up the compliant actuator. Values of tension in each cable are needed to calculate the linear elongation of cables, check if the system is between the tension limits, and calculate the torque required by the actuator.

A priori, the only known value is $\tau_{A}$, which is provided by the relation defined in Equation (4) by the linear sensor. Figure 7 represents the triangle of equilibrium of tensions, which can be deduced from the equilibrium of forces shown in Figure 6 by imposing the wrench closure in point $C_{i}$. As it was assumed that point ***C*** is massless, all tensions are coplanar in the plane Π. This equilibrium is expressed (considering unitary vectors) as:

(30) $$\tau_{B}{\left(\hat{C_{i}B\;}\right)}^{F}+\tau_{C}{\left(\hat{C_{i}D}\right)}^{F}+\tau_{A}{\left(\hat{C_{i}A}\right)}^{F}=0.$$

Angles α and β are calculated once the value of *θ* is obtained. The direction and sense of $\tau_{B}$ is known because it is coincident with the $l_{B}$ direction, so it only needs one more value, which can be the value of $\tau_{B},$ the value of $\tau_{C}$, or the direction of $\tau_{C}$ in plane Π. This last value was used in this analysis.

In order to obtain the direction of $\tau_{C}$ in plane Π, φ is obtained from geometric relations as:

(31) $$\varphi =\cos^{-1}\left(\frac{\vec{DC}\cdot \left(-\vec{BA}\right)}{\left\|\vec{DC}\|\|\vec{BA}\right\|}\right).$$

**Figure 8:**
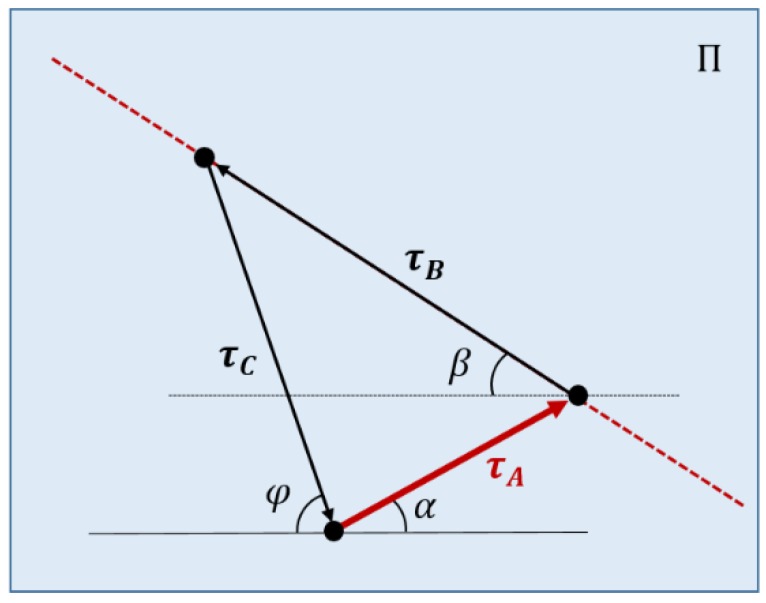
Equilibrium of forces at point $C_{i}$.

Knowing the direction of the tension $\tau_{C}$, all cable tensions can be obtained by imposing the equilibrium of wrenches as seen in Figure 8. The stiffness of each cable is:

(32) $$k_{i}=\frac{EA}{L_{i}}$$

where *E* is Young’s Modulus; *A*: is the section of the cable; and $L_{i}$ is the length of the cable. With the stiffness and cable tension, the longitudinal deformation can be obtained by using Equation (4).

In order to use Equation (2), the value of the external wrenches needs to be defined, which are responsible for the tension of the cables. Considering the separated wrench of $EE_{1}$ and $EE_{2}$, W is defined as:

(33) $$W^{F}=\left[\begin{array}{c} F\\ M_{o} \end{array}\right]=W_{EE_{1}}^{F}+W_{EE_{2}}^{F}.$$

Wrenches applied on both end effectors are considered from the geometric center of $EE_{1}$. The symmetry and constant density along its volume is considered in order to set the following external wrench:

(34) $$W_{EE_{1}}^{F}=\left[\begin{array}{c} -\left(m_{1}\right)g^{F}\\ 0 \end{array}\right].$$

As this is a kinetostatic analysis, $EE_{2}$ is static, so:

(35) $$G_{L}=\tau_{C_{L}}.$$

Wrenches applied on $EE_{2}$ have to be applied in a perpendicular direction of the rod:

(36) $$W_{EE_{2}}^{F}=\left[\begin{array}{c} G_{T}^{F}-\tau_{C_{T}}^{F}\\ R_{M}^{F}{\left(\vec{O^{\prime }D}\right)}^{M}\times (G_{T}^{F}-\tau_{C_{T}}^{F}) \end{array}\right].$$

The value of the wrench exerted in $EE_{1}$ is due to the mass of $EE_{1}$. The wrench exerted on $EE_{2}$ is due to the components of the mass of $EE_{2}$ perpendicular to the rod as no friction is considered between the cylindrical joint of ***D*** and the rod. The projection of $EE_{2}$’s weight over the rod is:

(37) $$G_{L}^{F}=T_{M}^{F}\left(G^{F}\cdot k^{M}\right).$$

The perpendicular projection of this weight is:

(38) $$G_{T}^{F}=G^{F}-G_{L}^{F}=mg^{F}-G_{L}^{F}.$$

**Figure 9:**
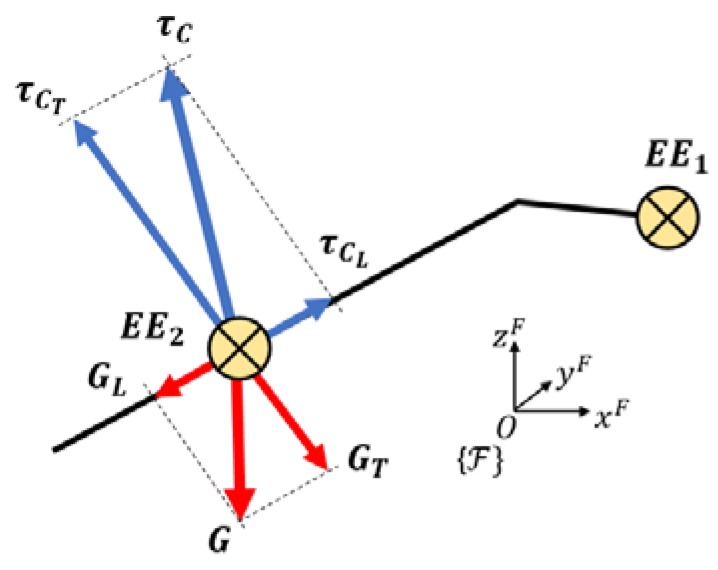
Wrenches applied to end effector 2.

The last step is to find the components of $\tau_{C}$ over the rod. The direction of $\tau_{C}$ is given by $\vec{C_{i}D}$ and the rod direction is given by $\vec{ED}$. The angle between $\vec{C_{i}D}$ and $\vec{ED}$ is:

(39) $$\cos\left(\hat{\vec{C_{i}D},\;\vec{ED}}\right)=\frac{\left(\vec{C_{i}D}\cdot \vec{ED}\right)}{\left\|\vec{C_{i}D}\|\|\vec{ED}\right\|}\;\tau_{C}=\frac{\;\tau_{CL\;}}{\cos\left(\hat{\vec{C_{i}D},\;\vec{ED}}\right)}$$

where $\tau_{CL}=G_{L}$ due to the neglecting friction and dynamic effects as seen in Figure 9.

## 7. Theoretical Results

A model of a robot with the compliant actuator was developed in the multi-body dynamic simulator MSC ADAMS. This software can obtain values that are difficult to measure, such as tension in cables, forces and reactions in all joints and bodies, and friction and elongation in pulleys and cables. In addition, multiple kinds of motions can be imposed in the actuators.

**Figure 10:**
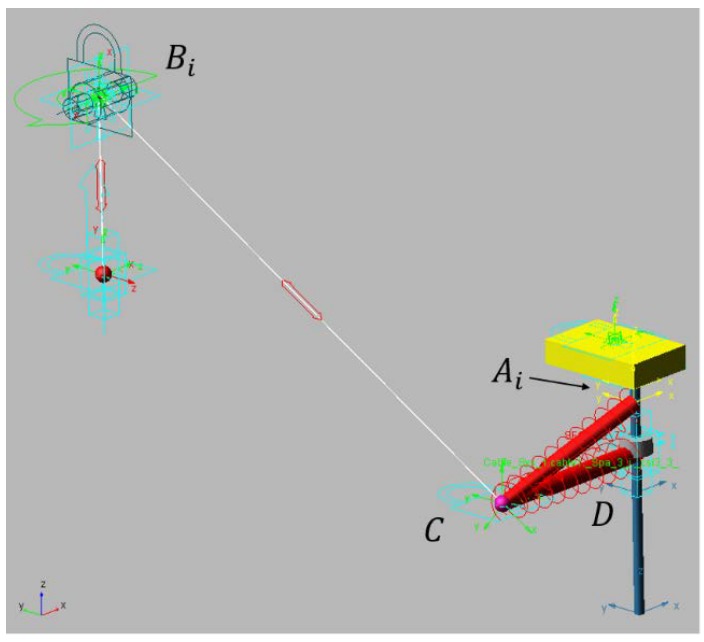
Model of the compliant actuator in the multi-body dynamic simulator MSC ADAMS.

In this model, linear and vertical motion were imposed on the end of the white cable shown in Figure 10. This cable passed along a pulley whose parameters are listed in Table 1. The cable was attached to point C, which was modeled with a negligible mass of 10^−2^ kg. Properties of the nylon cable modeled are shown in Table 2.

The $EE_{1}$ has been modeled as a rigid body fixed in space. As shown in Section 2, redundancy can provide this position even when the perturbation of the compliant actuator is exerted. The join between $A_{i}$ and *C* is the spring that adds the needed flexibility to make the linear sensor measurement more sensible to small tension variations in that branch of the cable. In D is the $EE_{2}$, which was modeled as a body with cylindrical restriction with the vertical rod fixed to $EE_{1}$. The cable between D and *C* was modeled with a spring of 222 mm to analyze its elastic behavior. Spring stiffness was obtained by using Equation (32) and the values from Table 2. The spring stiffness for this cable was 4.83 N/m.

Point C is a solid sphere of $10^{-2}$ kg. In $B_{i}$, it is situated in a pulley with a radius of $2.5\times 10^{-3}\;m$ that was able to rotate around the axis of its hole and on its vertical axis. In this model, the force was applied by exerting position control on the final part of the cable (the red ball in Figure 10). The proposed algorithm allowed us to find the position of D for each length of the cable. The cable was modeled with the parameters shown in Table 2.

The actuated cable was modeled with the values in Table 2. Elastic behavior and friction with the pulley was modeled; however, mass and inertia were neglected due to the reduced section of the cable. The modeling of the cable was a linear spring.

Table 3 shows the initial positions of each relevant part of the model. Point references and the reference frame can be seen in Figure 3. All values were measured from the fixed frame *O*.

$EE_{1}$ was fixed as well as the vertical rod to make possible the isolated analysis of the compliant actuator. $EE_{2}$, with 0.3 kg of mass, could move along the rod without friction with the unique actuation of cable *CD*, which is represented as a spring in Figure 10. In order to leave the end effector moving without attachment, we needed to know the forces that acted on it, which were provided by using the method proposed in this article. The simulated experiment, which is shown in graphs as the last 10 s, and each one of the 400 samples was taken each 0.025 s. The speed of the control cable ($l_{B}$) was approximately 18 mm/s (0.018 m/s). As can be seen in the following graphs, this speed was slow enough to neglect the dynamic effects.

**Figure 11:**
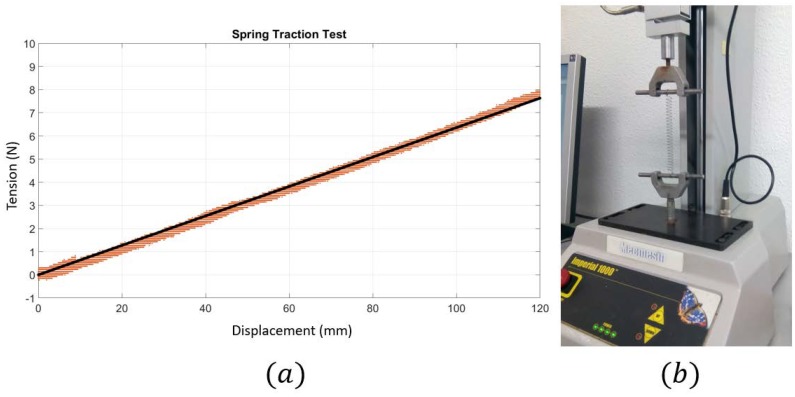
(**a**) Curve of the spring situated as a link between A and C: Tension versus elongation; (**b**) Device where traction test was done: Mecmesh Imperial 1000.

Finally, the sensorized cable *AC* was modeled as a spring. This spring stiffness was obtained by using a traction test of the spring as can be seen in Figure 11. The linear region of the spring was considered from 0 to 120 mm and its stiffness, considered constant, as $k_{A}=63.6\frac{N}{m}$. This maximum length for the spring imposes a restriction in the movement of $EE_{2}$. In that maximum elongation, the force achievable was 7.5 N (+2.2 N of the force of the linear encoder) = 9.7 N.

The linear sensor used was a LX_EP-40 of UniMeasure modeled as a constant force of 2.2 N compressing the spring. This force was measured and is also specified in the sensor datasheet. So, the stiffness of the link which connects A with C was modeled as a spring with an additional compressing force of 2.2 N. The maximum force achievable with springs was 7.5 N + 2.2 N = 9.7 N. At the beginning of the simulated experiment, the system was pre-tensed to begin in an equilibrium state where the tension in all cables had a non-zero value. This previous pre-tension mode was not shown because it was considered as not relevant data in this analysis.

A comparison between the simulation and calculated values of the method proposed in this article are shown below. In Figure 10, the position of $EE_{2}$ along the rod, in red, represents the simulated values and the values obtained with kinetostatic method are in black. The available length of the rod was 260 mm and the analysis was made from −100 to −270.

**Figure 12:**
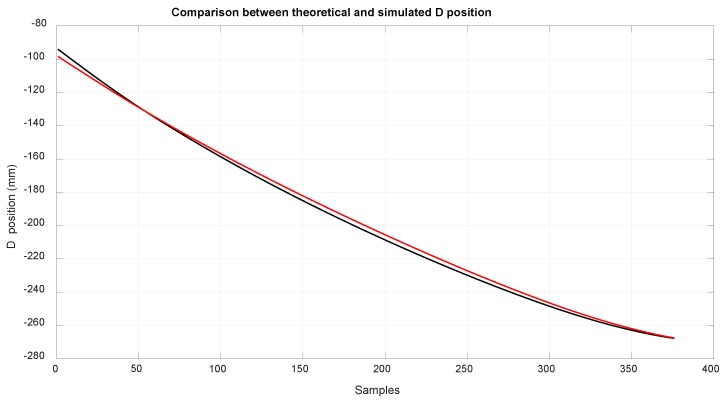
Comparison of the theoretical position of D (black) and the simulated position of D (red).

**Figure 13:**
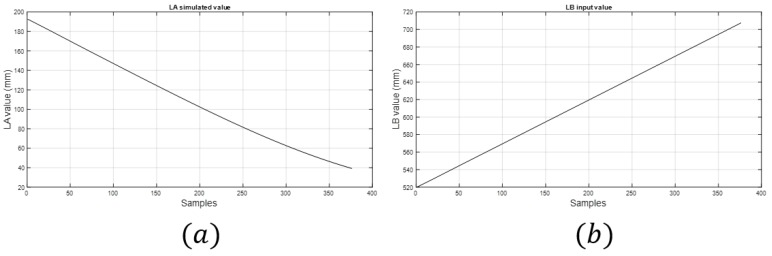
(**a**) Values of LA simulated that are considered in the kinetostatic method as the sensor value. (**b**) Input values of LB, length of controlled cable.

**Figure 14:**
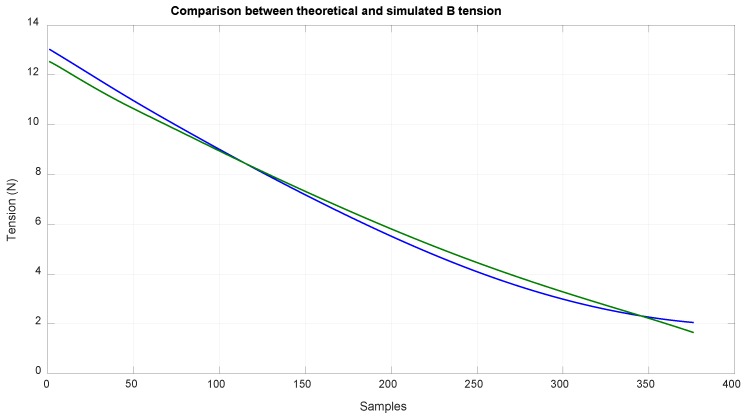
Comparison of the theoretical tension of B cable (blue) and the simulated tension of B cable (green).

**Figure 15:**
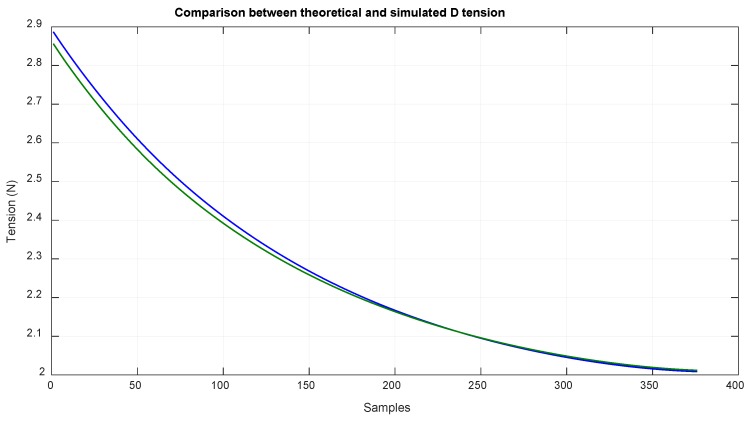
Comparison of the theoretical tension of D cable (blue) and the simulated tension of D cable (green).

The input values in the theoretical method were obtained by using data acquired in a simulation with MSC ADAMS. Figure 12 shows the value obtained of LD, and Figure 13 shows the value obtained of LB and LA, which is the measure of the linear sensor. Looking at the configuration of the system (Figure 3), it can be seen that it was difficult to reach positions close to $EE_{1}$ with $EE_{2}$, which was due to the high tension needed to reach them, and can likely be deduced by extrapolating the data from Figure 14 and Figure 15.

With this method, the tension values of the tree cables could be obtained. Figure 14 shows the tension values of cable B, which are related to the torque that the motor has to exert; additionally, this tension value is highly related to the deformation of this cable as the length is proportional to the cable deformation as seen in Equation (32).

In order to perform the first validation of these results, the error was measured between the simulated results and the results from this method. Table 4 shows the mean and standard deviation of the absolute error of each sample:

(40) $$\varepsilon_{a}={Value}_{calculated}-{Value}_{simulated}.$$

Table 5 considers the absolute imprecision values, which considered the absolute value in order to obtain the mean. The standard deviation with absolute value was also considered:

(41) $$Ea=\frac{\left|{Value}_{calculated}-{Value}_{simulated}\right|}{nº\;of\;samples}.$$

Table 6 shows the mean of the relative error between the calculated and simulated results with its value along all samples. This error is defined as:

(42) $$\varepsilon_{R}=\frac{\varepsilon_{A}}{{Value}_{simulated}}.$$

## 8. Experimental Results

In order to verify that the simulated model was valid, a prototype was built to compare the real world with the simulated results. The dimensions of the dynamic model in MSC ADAMS and the prototype are defined in Table 7 (values are considered in the reference frames shown).

**Figure 16:**
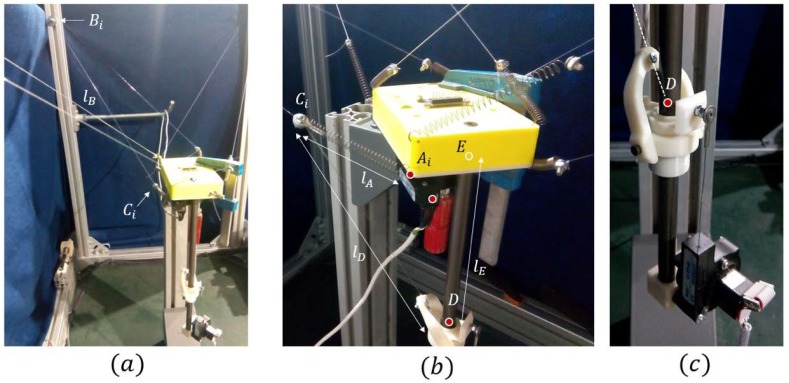
(**a**) General view of the CDPR where it shows point $B_{i}$ where the compliant actuator begins and the controlled length $l_{B}$; (**b**) $EE_{1}$ with a fixed position, with the compliant actuator coming from the left $l_{B}$ and split in $l_{D}$ attached to $EE_{2}$ and $l_{A}$. It can be seen that $l_{A}$ is made of a spring attached to $C_{i}$ and $A_{i}$ and a thin blue cable inside the spring coming from the linear encoder output (marked with a red dot); (**c**) $EE_{2}$ with the central axis of the rod marked with a red dot and the extrapolation of the cable marked with a white discontinuous line.

As seen in Figure 16c, $EE_{2}$ was formed by a mechanism of two degrees of freedom (rotations) that allowed the alignment of cable $l_{D}$ with the central axis of the rod. This was made to reduce the friction effects and to ensure that the $l_{D}$ cable always ended at the axis of the rod. The distance of $l_{D}$ was considered from $C_{i}$ until the red point and not only the physical cable length. The linear encoder of Figure 16b exerted 2.2 N of force to $EE_{2}$ due to the tension of its cable. This force was added to the $EE_{2}$ mass in the model.

**Figure 17:**
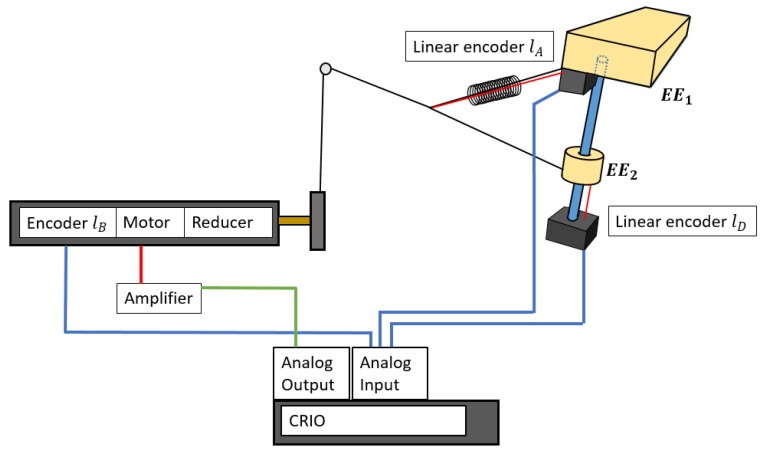
Scheme of the signals considered. Two linear encoders read the values of $l_{A}$ and $l_{D}$ while the value of $l_{B}$ is obtained by using the encoder of the motor. In blue are the lines of the incoming values from the sensors, in red are the power lines, and in green are the outcoming signals to control the motors. The processor was a CompactRIO 9081 with modules for analog input and output.

A scheme of the experimental setup is shown in Figure 17. The incremental linear encoder used was an LX-EP-40 of UniMeasure. It is able to measure the linear distance of the cable deployed from its base. The cable of this encoder was under a nominal tension of 2.2 N. The measured resolution was 2.45 ± 0.35% counts/mm, giving a precision of 0.4 mm/count. The sensing element of the encoder as an optical incremental encoder with electrical outputs consisting of two square wave, Transistor-Transistor Logic (TTL) output channels in quadrature. One of these encoders was used to find the value of $l_{A}$, as seen in Figure 16b, which is the encoder that will be situated in the robot in normal duty. The second linear encoder was used to measure the distance of the $EE_{2}$ from E, denominated as $l_{D}$, and check if the real position corresponded with the theoretical one.

The length of the cable was measured by using the encoder of the motor. This motor coiled the cable around a pulley of 14 mm in diameter that was attached to the shaft of the motor. The motor was an RE 13 Graphite Brushes motor of 3 Watts of Maxon that provided a nominal torque of $2.42\times 10^{-3}$ nm. The motor had a reducer of 275:1, so the nominal torque in the shaft of the motor was 0.67 nm. As the pulley where the cable was coiled had a diameter of 14 mm, the nominal tension exerted to it had a value of 95 N. The motor obtained its power from the amplifier ADS 50/5 4-Q-DC of Maxon, which applied a current control of the motor.

The encoders of the actuators were attached to the shaft of the motor before the reduction. These encoders were of the MR Type S, 16 CPT, 2 Channels of Maxon. They detect 16 counts per turn of the motor, or 16 × 275 = 4400 counts per turn of the shaft of the entire actuator. Each count appears after 0.08°, which corresponds in the linear displacement of the cable to one count each of $9.7\times 10^{-6}$ m.

The precision provided by the encoders was enough to work with an accuracy in the experiment of 0.5 mm.

All of the signals were handled by using a CompactRIO 9081 from National Instruments that had a central processing unit (CPU) Dual-Core 1.06 GHz, 2 GB DRAM, 16 GB storage, and an Field-Programmable Gate Array (FPGA) Virtex-6 LX75. The modules used were the analog input module AI-NI9205 and the analog output module AI NI-9264.

The absolute accuracy of the analog input module was $3.23\times 10^{-3}$ V with random noise of $2.4\times 10^{-4}$ Vrms and a sensitivity of $9.6\times 10^{-5}$ V. The calibration of this module was done by using the self-calibration software. However, the signals acquired by this module were TTL signals from the encoders, so it did not need more accuracy than this kind of signal requires.

The analog output module requires more precision as this voltage is responsible for controlling the current of the actuator. This module had a resolution of 16 bits, the gain error for this experiment was $8\times 10^{-3}$ V, and the offset error was $10^{-2}$ V. Those values were enough to set the current value of the motor.

**Figure 18:**
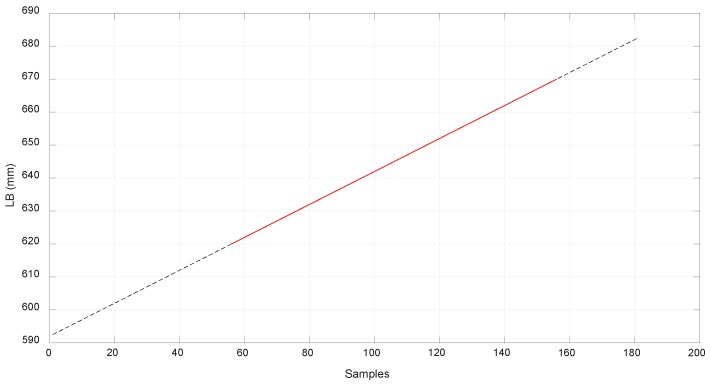
Comparison of simulated value of and real world results for $l_{B}$.

**Figure 19:**
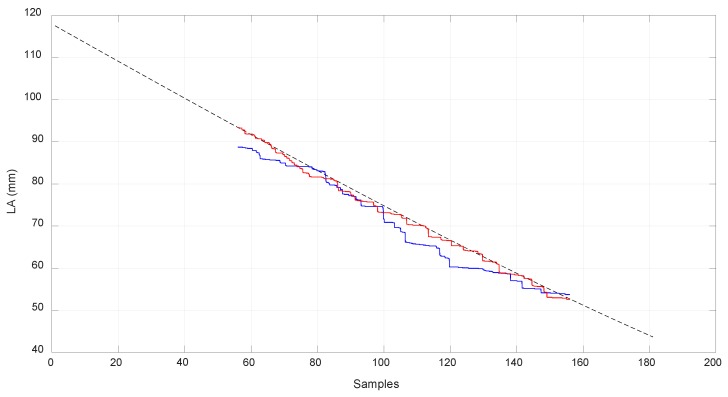
Comparison of the simulated and real world results for $l_{A}$. The dotted line corresponds to the simulated results with MSC ADAMS. The red line corresponds to the value of $l_{A}$ in an experiment where $EE_{2}$ goes up from its lower position. The blue line corresponds to the value of $l_{A}$ in an experiment where $EE_{2}$ goes down from its upper position.

**Figure 20:**
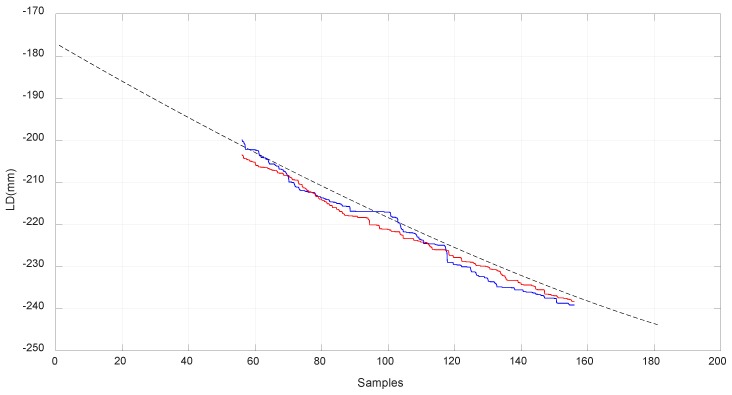
Comparison of the simulated and real world results for $l_{D}$. The red line corresponds to the value of $l_{D}$ in an experiment where $EE_{2}$ goes up from its lower position. The blue line corresponds to the value of $l_{D}$ in an experiment where $EE_{2}$ goes down from its upper position.

In Figure 18, Figure 19 and Figure 20, the values obtained for LB, LA, and LD with the MSC ADAMS simulation are compared with the values obtained from the real prototype. In order to make an appropriate comparison, the samples of the real values of $l_{B}$ were situated over the corresponding samples of the simulated values, which were from sample 56 to sample 156. The same correspondence of the samples considered the values of $l_{A}$ and $l_{D}$ and had the same scale for the comparison. The values of $l_{B}$ were obtained by a Proportional-Integral-Derivative (PID) control loop in the actuator where the value of $l_{B}$ acts as feedback.

Due to limitations in the prototype, only a limited range of movement was achieved. In order to reach higher positions with $EE_{2}$, longer springs are needed as the required values of $l_{A}$ are high when $EE_{2}$ is situated at a higher position than −200 mm as seen in Figure 13. As seen before, the range of linear stiffness of the spring was from 0 to 120 mm so the values corresponding to that range are shown in Figure 19 in red color. In order to focus this analysis in terms of the behavior of the compliant actuator, $EE_{1}$ was attached to the fixed frame.

We conducted t experiments to calculate the repeatability and statistical distributions. Two experiments were undertaken by displacing the $EE_{2}$ from its lower position to the upper position and another two experiments were developed by displacing the $EE_{2}$ from its upper position to the lower position. This method should be valid for the two types of displacements so the two measures were considered together as experiments with the same working conditions to obtain the statistical distributions.

**Figure 21:**
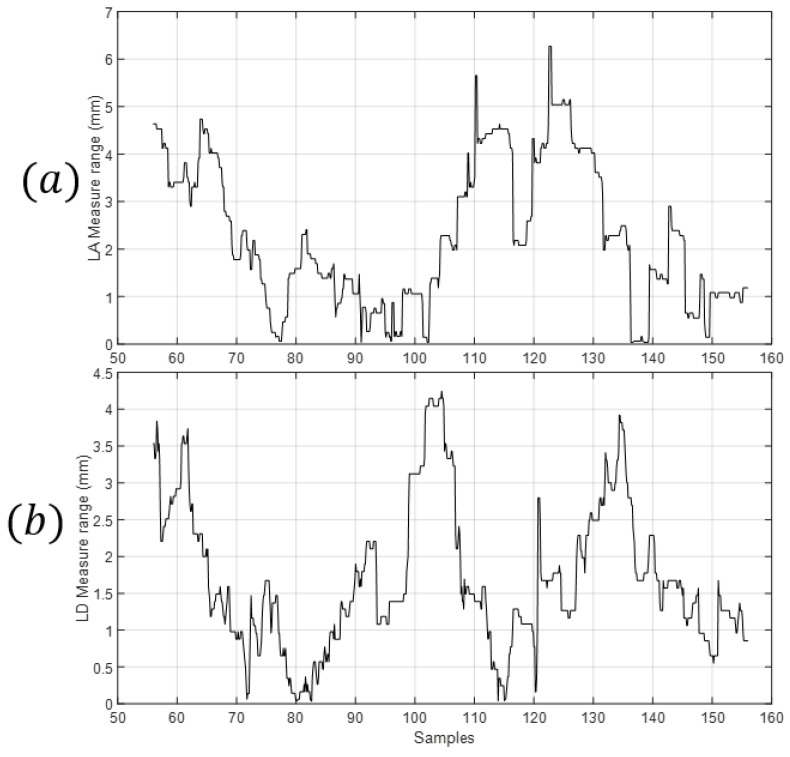
Average range of the measured values for calculating the repeatability. (**a**) From the two measures of $l_{A}$; (**b**) From the two measures of $l_{D}$.

The repeatability of the measures was based on the method presented in [44]. The values of the range of the measures can be seen in Figure 21a,b.

Two different measures from different experiments were performed. The maximum value of the range of the measure of $l_{A}$ was 6.3 mm while the mean value of its range was 2.2 mm. The maximum value of the range of the value of $l_{D}$ was 4.2 mm while the mean value of its range was 1.7 mm. The standard deviation due to repeatability is calculated and the results are shown in Table 8.

**Figure 22:**
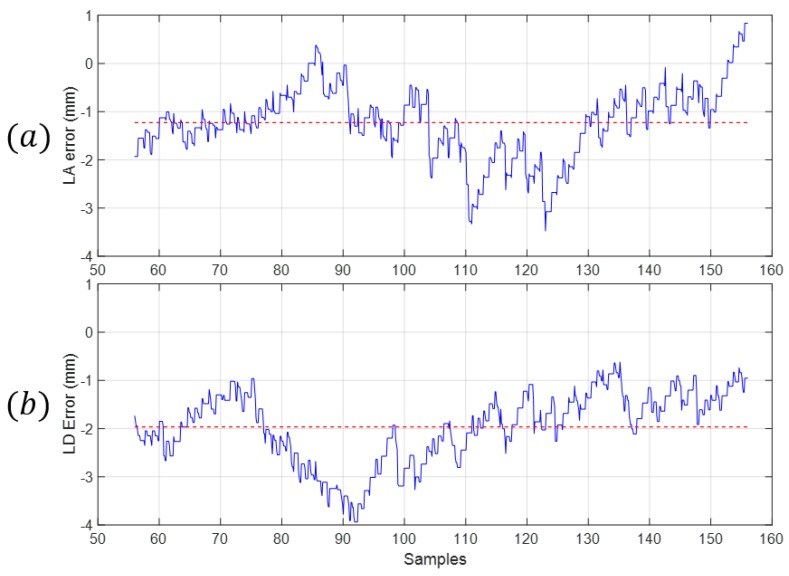
The blue graph is the error between the simulated value and the real value. The red dotted line is the mean value of the error between the real measure and the simulated one. (**a**) Values of the measure of $l_{A}$; (**b**) Values of the measure of $l_{D}$.

Measuring the difference between the real values and the simulated ones allows for the visualization of the deviation of the measurements from the desired values. The maximum error between the real and simulated measures is shown in Figure 22 and Table 9 as well as the mean error along the whole range of the mechanism.

The range of $l_{A}$ was [54, 94] mm and the range of $l_{B}$ was [−237, −202] mm. The relative error comparing the mean error with the range of each measure is shown in Table 10.

## 9. Discussion

The method presented in this article gives the position of the second body of the end effector and tension of all parts of the compliant actuator with limited accuracy for any given value of $l_{B}$. The following values are needed as input:

- Position and shape of the first end effector.
- Position of the points where cables are deployed from the fixed structure.
- Characteristics of the cable, spring, and linear sensor.
- Mass of the two end effectors.
- Constant length of the cable attached to the second end effector.
- Variable length of control cable ($l_{B}$) and measure of the linear encoder as the input of the algorithm.

The analysis proposed was kinetostatic and dynamic effects were neglected. This consideration is valid when slow movements are made. In this case, $l_{B}$ moved with a speed of 18 mm/s (0.018 m/s), which, as seen, was low enough to neglect the dynamic effects.

The number of samples considered was 400 samples in the simulated experiment and 8000 in the real world experiment. In each sample, the mathematic method ran with only one iteration of the algorithm shown in Figure 5. It can be seen that with only one iteration, the absolute imprecision of $EE_{2}$’s position was below 3.5 mm of error when compared with the simulated model.

The results obtained with the analysis shown in this study allows for the creation of the control position of a cable-driven parallel robot with two end effectors configured in the way defined with a cable that exerts its effort over the two bodies. Considering this methodology of measuring with a linear encoder in the intermediate links, it could be possible to increase the number of end effectors and complexity of the system.

In addition, by using the information provided by the sensor and the spring attached to it, it is possible to calculate tension in all cables. Knowing the tension in control cable ($l_{B}$), it is easier to develop better joint control of the actuator by adding this variable tension to the control scheme. Tension in cable $l_{D}$ can be useful in those cases where there is interaction with humans or delicate environments to restrict the cable tension to increase security. The value of tension of cable $l_{A}$ allows for the control of $EE_{1}$ due to its direct influence being one of the wrenches applied by the n cables.

The dynamic model used in MSC ADAMS simulated a totally controlled environment to test the validity of this algorithm. It can be seen that the error between both results is acceptable for slow applications that do not need high precision or high speed, such as underwater robots or rehabilitation devices.

The dynamic model of MSC ADAMS was validated with real-world experiments developed with a prototype with the same configuration as the dynamic model. There were some sources of error between the prototype and the model, such as the friction effects not modeled, deviation of the cable of the linear encoder before getting into the spring, or the presence of electric noise in some measurements.

Several hypothesis were imposed in order to make this analysis possible:

- Mass of C was neglected.
- Mass of cables was neglected.
- Pulleys’ geometry and their dynamics were not considered.

The mass of point C was very small because in the actual prototype it is made by a system of bolts and nuts of low weight. In the simulated model, this weight was considered to be 10^−2^ kg. This hypothesis was crucial in this analysis to work with one plane Π.

Nylon cables have a density of 1150 (kg/m^3^) and an area of 0.28 mm^2^. For a length of 700 mm, the cable weight is 0.22 g, which in this analysis could be easily neglected. With this hypothesis, the catenary effects were not considered.

It is noticeable that the mean error between the real and simulated measures had a negative value for the position of the second body of the end effector as well as a negative value for the elongation of the spring with the sensor. This effect could have appeared due to a non-precise characterization of the elastic effects of the nylon cable that in the real case could have a lower stiffness than in the theoretical and simulations. The Young’s modulus of the cable has been defined in Table 8 because is the parameter related to nylon. However, the behavior of the nylon varies with the use and other conditions as seen in [44]. In addition, the neglecting of the mass of the cables and the mass of C could have relevance in the appearance of that error. In [37], different sources of errors are explained when an elasto-geometrical model is used with cable mechanism. Similarities between the graphs presented in both articles are easily detected.

One way to reduce this error could be the consideration of a cable stiffness of lower values than the theoretic value of the nylon and the consideration of the cable mass.

Finally, the dynamics and geometry of the pulleys were considered in the dynamic simulator; however, the algorithm considered them as points in their center.

Future research should consider kinetostatic and dynamic analysis of this kind of robot in order to improve control with higher speeds.

## 10. Conclusions

In this paper, we designed a novel cable-driven robot with a reconfigurable end effector by using a “compliant actuator” setup. The proposed design was simulated within MSC ADAMS with a range of movement of the end effector mechanism of 170 mm with an 0.63% of error with respect to the theoretical results.

The experimental tests validated the simulated model with a 5.4% error, which can be seen as suitable for the intended application due to the intended human–robot collaboration and the high elastic effects present in the robot.

This compliant actuator mechanism is open to a wide range of applications related to end effectors with many dof where they are controlled by using the redundancy in the external actuators.

## Figures and Tables

**Table 1:** Pulley parameters.

Parameters	Values
Diameter (mm)	5
Width (mm)	4
Depth (mm)	0.5

**Table 2:** Cable parameters.

Parameters	Values
Diameter (mm)	0.6
Young ^1^ (Pa)	3.9 × 10^9^
Density (kg/m^3^)	1150

^1^ Young’s Modulus.

**Table 3:** Position of relevant parts.

Parameters	X (mm)	Y (mm)	Z (mm)
$O^{\prime }$	355	477	607
$\;A_{i}$	310	377	575
*D*	355	417	502
*C*	193	358	642
$B_{i}$	23	953	902

**Table 4:** Absolute error between the model and simulation.

Parameters	Values	Parameters	Values
Mean error LD	−1.53 mm	Std. Dev. ^1^ LD	1.71 mm
Mean error $\tau_{B}$	−0.05 N	Std. Dev. ^1^$\;\tau_{B}$	0.27 N
Mean error $\tau_{D}$	0.01 N	Std. Dev. ^1^$\;\tau_{D}$	0.01 N

^1^ Standard Deviation.

**Table 5:** Absolute imprecision between model and simulation.

Parameters	Values	Parameters	Values
Mean error LD	2.09 mm	Std. Dev. ^1^ LD	0.95 mm
Mean error $\tau_{B}$	0.24 N	Std. Dev. ^1^$\;\tau_{B}$	0.13 N
Mean error $\tau_{D}$	0.01 N	Std. Dev. ^1^$\;\tau_{D}$	0.01 N

^1^ Standard Deviation.

**Table 6:** Relative error between model and simulation.

Parameters	Values
Mean error LD	0.63%
Mean error $\tau_{B}$	−1.74%
Mean error $\tau_{D}$	0.34%

**Table 7:** Dimensions of the robot.

Parameters	Values
$O\prime^{F}$ (mm)	[355, 447, 622]
$A^{M}$ (mm)	[−45, −70, −30–17] ^1^
$B^{F}$ (mm)	[23, 953, 902]
$E^{M}$ (mm)	[0, −30, −30]
$m_{EE}{}_{1}$ (Kg)	0.4
$m_{EE}{}_{2}$ (Kg)	0.2
$l_{D}$ (mm) ^2^	222

^1^ Position of linear encoder is 17 mm under the vertex of the body; ^2^ Length of cable between C and D when not under tension.

**Table 8:** Range of the measuring.

Parameters	$l_{A}\;(\mathrm{mm})$	$l_{D}\;\;(\mathrm{mm})$
Max. range	6.2	4.2
Mean range	2.2	1.7
Max. Std. Dev ^1^	5.5	3.7
Mean Std. Dev ^1^	1.9	1.5

^1^ Std. Dev.: Standard Deviation

**Table 9:** Deviation from simulated and real measures.

**Parameters**	$l_{A}$ **(mm)**	$l_{B}$ **(mm)**
Max. error	−3.5	−3.9
Min. error	0	−0.6
Mean error	−1.2	−1.9

**Table 10:** Relative error between simulation and real prototype.

Parameters	Values
Mean error LA	3.0%
Mean error LD	5.3%
